# Single cell studies of mouse embryonic stem cell (mESC) differentiation by electrical impedance measurements in a microfluidic device

**DOI:** 10.1016/j.bios.2016.02.069

**Published:** 2016-07-15

**Authors:** Ying Zhou, Srinjan Basu, Ernest Laue, Ashwin A. Seshia

**Affiliations:** a Nanoscience Centre, Department of Engineering, University of Cambridge, 11 JJ Thomson Avenue, Cambridge CB3 0FF, United Kingdom; b Department of Biochemistry, University of Cambridge, 80 Tennis Court Road, Cambridge CB2 1GA, United Kingdom

**Keywords:** Microfluidics, Single cell trapping, Impedance spectroscopy, Stem cells, Heterogeneity

## Abstract

Biological populations of cells show considerable cell-to-cell variability. Study of single cells and analysis of cell heterogeneity are considered to be critical in understanding biological processes such as stem cell differentiation and cancer development. Recent advances in lab-on-a-chip techniques have allowed single-cell capture in microfluidic channels with the possibility of precise environmental control and high throughput of experiments with minimal usage of samples and reagents. In recent years, label-free techniques such as electrical impedance spectroscopy have emerged as a non-invasive approach to studying cell properties. In this study, we have designed and fabricated a microfluidic device that combines hydrodynamic trapping of single cells in pre-defined locations with the capability of running electrical impedance measurements within the same device. We have measured mouse embryonic stem cells (mESCs) at different states during differentiation (*t*=0 h, 24 h and 48 h) and quantitatively analysed the changes in electrical parameters of cells during differentiation. A marked increase in the magnitude of the cell impedance is found during cell differentiation, which can be attributed to an increase in cell size. The analysis of the measurements shows that the nucleus-to-cytoplasm ratio decreases during this process. The degree of cell heterogeneity is observed to be the highest when the cells are at the transition state (24 h), compare with cells at undifferentiated (0 h) and fully differentiated (48 h) states. The device enables highly efficient single cell trapping and provides sensitive, label-free electrical impedance measurements of individual cells, enabling the possibility of quantitatively analysing their physical state as well as studying the associated heterogeneity of a cell population.

## Highlights

• Microfluidic device combines hydrodynamic trapping and impedance spectroscopy.
• Mouse embryonic stem cells trapped and monitored during the differentiation cycle.
• A marked variation in cell electrical parameters is observed during differentiation.
• The degree of cell heterogeneity during differentiation is quantitatively analysed.
• Highly sensitive label-free measurements of electrical cell markers demonstrated.

## 1 Introduction

Biological populations of cells show considerable cell-to-cell variability either as a result of the intrinsic stochasticity of gene expression or due to extrinsic factors such as subtle differences in cell microenvironments (Haselgrubler et al., 2014, Huang, 2009). There has been much interest recently in understanding how this non-genetic heterogeneity affects how single cells within a population respond to stimuli and whether some cells act differently to others. The most common tools currently available to study cell heterogeneity are automated imaging of large numbers of cells (followed by complex data analysis) or flow cytometry where the scatter or total fluorescence of single cells can be measured (Huang, 2009). Flow cytometry gives the statistical distribution of a population at the resolution of individual cells and provides the most lucid information on population heterogeneity. However, flow cytometry cannot monitor temporal changes in the properties of an individual cell. To monitor and track single cells in real time, techniques such as live video-microscopy have been developed and used. Single-cell analysis offers the opportunity to study the kinetic changes of individual cells over time, which cannot be obtained from flow cytometry.

Recently, there has been considerable effort put into developing label-free approaches that do not require complex equipment such as electric cell-substrate impedance sensing (ECIS), single-cell dielectric and impedance spectroscopy (Bagnaninchi and Drummond, 2011, Chen et al., 2014, Gawad et al., 2004, Holmes et al., 2009, Morgan et al., 2007, Myers et al., 2013, Song et al., 2013, Zhu et al., 2014). Such methods are unbiased, allowing the identification of cells for which the expression of specific markers are unknown. Electric cell-substrate impedance sensing (ECIS) has been used for decades as a label-free real-time way of monitoring a range of cellular processes that involve changes in cell shape or size such as cell growth, division, adhesion and apoptosis. However, ECIS is mostly carried out on bulk populations of cells and thus cannot provide information on individual cells and cell–cell variations (heterogeneity). Moreover, cell–cell interactions, which are inevitable in bulk measurements, can influence measurement results and complicates direct interpretation of the data.

Recent advances in micro-/nanofabrication and lab-on-a-chip techniques have allowed single-cell impedance spectroscopy, i.e., single-cell impedance flow cytometry (Gawad et al., 2001, Holmes et al., 2009, Malleo et al., 2010, Sun and Morgan, 2010), opening up the possibility of studying single cells and cell-to-cell heterogeneity. These studies have not only allowed the distinction of different cell type subpopulations within a mixed sample (Gawad et al., 2001, Holmes et al., 2009), but also simplified analysis such that it is now possible to extract parameters that describe the cell such as cell size, membrane capacitance and cytoplasm conductivity (Asami, 2002, Gawad et al., 2004). However, similar to fluorescence-based flow cytometry that provides population snapshots and cannot monitor temporal changes within individual cells (Huang, 2009), impedance-based flow cytometers also lack the ability to track time-dependent changes in properties of individual cells (Malleo et al., 2010).

As a result, there has been much interest recently in developing techniques for single cell trapping, manipulation and analysis. One of these techniques, which does not require complicated instrumentation, is hydrodynamic trapping. Various designs have been proposed to realise single-cell trapping using hydrodynamic forces (Bell et al., 2011, Di Carlo et al., 2006, Tan and Takeuchi, 2007, Tan and Takeuchi, 2008). Among these, a novel trapping design described by Tan et al., based on differential fluidic resistances in microfluidic channels, has been associated with efficient and reliable trapping of micron-scale particles and cells (Tan and Takeuchi, 2007, Tan and Takeuchi, 2008). Hydrodynamic trapping provides great opportunities for time-dependent single cell study. An example of coupling cell capture with impedance analysis has been illustrated by Malleo et al., who have demonstrated impedance study of HeLa cells in response to chemical disruption in a microfluidic device containing multiple trapping sites (Malleo et al., 2010).

Analysis of stem cells at single-cell level is critical when understanding processes such as stem cell differentiation and cancer development. Methods have been developed to study stem cells and understand how the properties of stem cells change during differentiation (Bagnaninchi and Drummond, 2011, Flanagan et al., 2008, Myers et al., 2013, Pethig et al., 2010, Song et al., 2013). Real-time monitoring of stem cell differentiation is made possible by using the bulk ECIS technique and unique changes in impedance that correlate with differentiation are observed (Bagnaninchi and Drummond, 2011). However, bulk measurements cannot provide information about individual cells or cellular heterogeneity. On the other hand, single cell analysis using impedance flow cytometry has also been carried out recently and showed that by looking at the electrical impedance ratio between two specific frequencies (a term called opacity), it is possible to distinguish undifferentiated and differentiated stem cells (Song et al., 2013). However, as mentioned before, flow cytometry only provides the instant information about cell distributions within a population and does not provide the time-dependent information on individual cells. Combining the real-time impedance measurement capability with single-cell analysis techniques is thus of great interest. Further, there is still lack of research currently in quantitatively studying electrical properties of individual stem cells and the cell differentiation process based on the label-free impedance-based methods.

In this work, we have combined the technique of hydrodynamic trapping together with impedance spectroscopy within a single microfluidic device that not only enables efficient trapping of single cells, but also allows electrical impedance-based monitoring of individual cells in a label-free, non-invasive and non-contact manner. We have performed impedance measurements for mouse embryonic stem cells (mESCs) at single-cell level and applied this technique to study the embryonic stem cell differentiation process. A very clear distinction between the cells at various differentiation states and a change in cell heterogeneity during differentiation has been observed. To our knowledge, this is the first time anyone has performed quantitative analysis of the embryonic stem cell differentiation process at single-cell level, using the electrical impedance-based approach. The electrical frequency response of individual stem cells during the course of differentiation has been studied for the first time, and electrical parameters of stem cells at different differentiation states have been extracted and quantitatively analysed. The results show that these extracted parameters, considered as electrical markers of cells (i.e., physical markers from electrical measurements), can be used to quantify their physical state.

## 2 Materials and methods

### 2.1 Design and theory

**Fig. 1:**
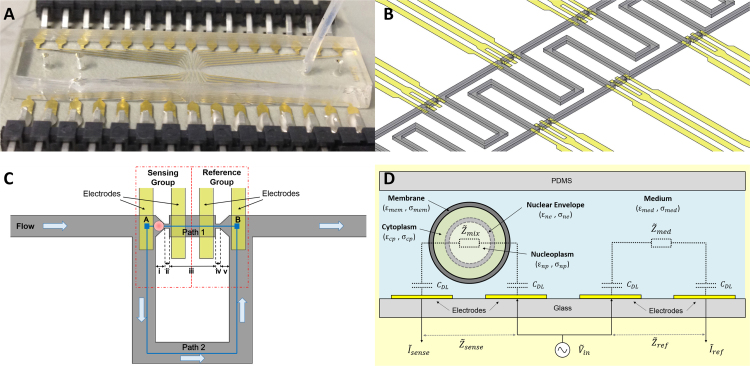
Device overview, structure and working principle. (A) A fabricated microfluidic device, composed of a PDMS top layer and a glass substrate with patterned electrodes. (B) Magnified 3D schematic diagram of the cell trapping channels (grey colour) and impedance sensing electrodes (yellow colour). (C) Schematic diagram illustrating the trapping and sensing mechanism. The channels are designed in such a way that the flow resistance along the short trapping path (Path 1) is lower than that of the U-shaped bypass channel (Path 2). When the trap is empty, a cell in the flow will be driven into the trap. Once the trap is occupied by a cell, the flow through Path 1 is blocked, and hence the next cell will be driven into the bypass channel and enter the next available trap. (D) Electrical model of a cell suspended in a medium inside the microfluidic system. (For interpretation of the references to color in this figure legend, the reader is referred to the web version of this article.)

An overview of the fabricated device is shown in Fig. 1A. Coplanar electrodes are patterned on the bottom glass substrate for impedance sensing. The top layer, made of PMDS, contains microfluidic trapping channels for single cell capture. Fig. 1B is a 3D schematic diagram showing the microfluidic channels (grey colour) as well as impedance sensing electrodes (yellow colour). Fig. 1C is the diagram schematic of one sensing unit, illustrating the mechanism of trapping and impedance sensing of a single cell. The principle of the cell trapping utilised in this work is based on the hydrodynamic trapping mechanism and the differential fluidic resistance exhibited in channels (Tan and Takeuchi, 2007, Tan and Takeuchi, 2008). The device is designed in such a way that differential electrodes are adapted into the microfluidic chip for electrical impedance spectroscopy. There are two paths from point A to point B: Path 1 and Path 2. The straight channel, Path 1, can be subdivided into five regions (notated as i, ii, iii, iv, v in Fig. 1C) based on different channel widths and geometries. Region i and v are the actual positions where cells will be physically trapped, so these two regions are referred to as “traps” throughout the text and face opposite each other. Only one of these traps will be normally occupied depending on the flow direction. The narrowest regions in Path 1 (ii and iv), known as the trapping gaps, are smaller than cells and thus allow cells to be mechanically constrained and immobilised in place in the traps (region i and v). Path 2 is a bypass channel, which shunts cells away from an occupied trap and leads them onto the next one. The flow resistance along Path 1 is designed to be lower than that of Path 2, so that a particle can be driven into a trap by hydrodynamic forces when the trap is empty. Once the trap is occupied by a cell, the flow through Path 1 is blocked, and thus the next cell will be driven into the bypass channel and enter the next available trap. The design and derivation details of the channel dimensions are described in Supplementary Information and summarised in Table S1.

As shown each of the adjacent cell traps has two corresponding impedance-sensing electrodes located underneath the channels. Depending on the flow direction, a cell can be captured in one of the traps. No matter which trap is occupied, the other one is always kept empty and can thus serve as a reference. This trapping configuration enables the use of a differential impedance measurement scheme. Differential measurements can eliminate any unexpected change and drift caused by the surrounding environment or electrode properties (Gawad et al., 2001, Malleo et al., 2010). Since the two traps are very close to each other, they have very similar conditions inside the channel, in terms of temperature, pH, conductivity, etc. Any common-mode effect due to any of these factors can be effectively rejected by using this differential design. This is particularly beneficial for single cell characterisation where high sensitivity and accuracy are required. As shown in Fig. 1C, the two electrodes underneath the occupied trap sense the impedance of the cell being captured, and thus are referred to as the sensing electrodes (or sensing group). The two electrodes underneath the empty trap located nearby measure the impedance of medium, and are thus called the reference electrodes (or reference group).

When an AC electric field is applied to a cell in suspension, the dielectric properties of the cell change as a function of frequency, known as Maxwell-Wagner dispersion. The dielectric behaviour of cell suspensions can be analysed using Maxwell′s mixture theory. Fig. 1D presents the electrical model of a cell suspended in a medium inside a microfluidic system. It should be noted that the scenario as depicted in this figure is idealised. In reality the electric field and current density distributions are non-uniform because of the structured channel with small trapping gaps. It has been verified from numerical simulations (COMSOL Multiphysics 4.4) that, in this design, the current density is concentrated (and thus highest) in the narrow trapping gap (Fig. S1 in Supplementary Information). The cell is modelled by the “double-shell” model (Asami et al., 1989, Irimajiri et al., 1978, Irimajiri et al., 1979), where the cell is composed of four phases, i.e., cell membrane, cytoplasm, nuclear envelope and nucleoplasm. The “double-shell” implies the thin cell membrane and nuclear envelope, with the membrane separating the cytoplasm from the ambient medium and the nuclear envelope separating the nucleoplasm from the cytoplasm. Each phase is modelled with its own electrical properties (conductivity ‘σ’ and permittivity ‘ε’). The conductivity and permittivity of the medium are $\sigma_{\mathrm{med}}$ and $\varepsilon_{\mathrm{med}}$ respectively. The double-shell model can be used for studying cells with a high nucleus-to-cytoplasm (N/C) ratio such as stem cells, as the properties of the nuclear envelope and nucleoplasm are taken into account. The total complex impedance of a cell surrounded by a medium (i.e., cell-medium mixture) in the sensing volume is ${\tilde{Z}}_{\mathrm{mix}}$ and the complex impedance of the medium in the reference volume is ${\tilde{Z}}_{\mathrm{med}}$. Coplanar electrodes are patterned on the bottom glass substrate. Between the electrode surface and the electrolyte forms an electrical double layer, of which the capacitance is notated as ${\tilde{C}}_{\mathrm{DL}}$. With double layer capacitance taken into account, the total impedance measured from the electrodes in sensing group is ${\tilde{Z}}_{\mathrm{sense}}$ and the total impedance measured from the electrodes in the reference group is ${\tilde{Z}}_{\mathrm{ref}}$. The expressions of these complex impedances, as a function of the electrical parameters (e.g., permittivity, conductivity) of each cell phase, are provided in Supplementary Information, based on Maxwell′s theory of interfacial polarisation. The differential spectrum of a cell can be obtained by normalising the impedance of the sensing group with regard to the impedance of the reference group (Malleo et al., 2010), i.e., ${\tilde{Z}}_{\mathrm{diff}}={\tilde{Z}}_{\mathrm{sense}}/{\tilde{Z}}_{\mathrm{ref}}$.

### 2.2 Device fabrication

Electrodes (20 nm Ti and 100 nm Au) were patterned on Pyrex glass wafers by lift-off. The master moulds for the microfluidic channels (geometric dimensions are listed in Supplementary Information Table S1) were fabricated using negative photoresist SU-8 2015 (MicroChem) by standard photolithography. Microfluidic trapping channels were then fabricated using PDMS (polydimethylsiloxane) soft lithography techniques. The fabricated device was connected to external instrumentation by surface mount connectors. The connectors were bonded to the electrode pads on the chip using silver conductive epoxy (Fig. 1A). Details of device fabrication are presented in Supplementary Information.

### 2.3 Cell preparation

Mouse embryonic stem cells were cultured in standard serum and LIF conditions as previously described (Reynolds et al., 2012). These cells were then trypsinized and resuspended in PBS for live cell impedance measurements. Fixed cells were prepared in 2% methanol-free formaldehyde (ThermoScientific, 28908) for 5 min and then resuspended in PBS for impedance measurements. Cell nuclei were prepared after fixation by incubating for 30 min on ice (with inversion every 10 min) in 10 mM Tris–HCl pH 8.0, 10 mM NaCl, 0.2% NP-40 and a protease inhibitor cocktail (Roche). Differentiated cells were prepared by withdrawal of LIF from the media for 24 and 48 h as previously described (Reynolds et al., 2012). These cells were then fixed and resuspended in PBS for impedance measurements.

### 2.4 Experiment and simulation

The microfluidic device was connected to a 1 ml syringe with PTFE tubing (0.59 mm ID × 0.25 mm Wall) and 23 G needles. Fluid flow was controlled by syringe pumps. Before use, the channels were pre-treated with 1% BSA (in 1×PBS) for 30 min to block hydrophobic interactions between biological samples and PDMS surface. The chip was connected with an impedance analyser (Solartron SI 1260) for impedance measurements. Prior to cell characterisation, the device was first filled with PBS buffer, and a calibration experiment was performed for the device itself, serving as a baseline for further cell measurements. After the calibration, cells were loaded into the device at flow rate of 20 µl/hr. Once all traps were occupied by cells (inspected with microscope), the device was washed with buffer. Cell impedance measurements were then conducted with the impedance analyser. A 100 mV input was used. The frequency range was from 100 Hz to 20 MHz, with 10 points being measured per decade. During the impedance measurements, the fluid flow was stopped to minimise the cell deformation caused by the fluid shear stress. No cell was observed to escape from the traps as long as the fluid connections (e.g., syringes, tubings) were kept undisturbed. Analytical simulations based on the double-shell cell model were performed using Matlab.

## 3 Results and discussion

### 3.1 Single cell trapping and impedance measurements

**Fig. 2:**
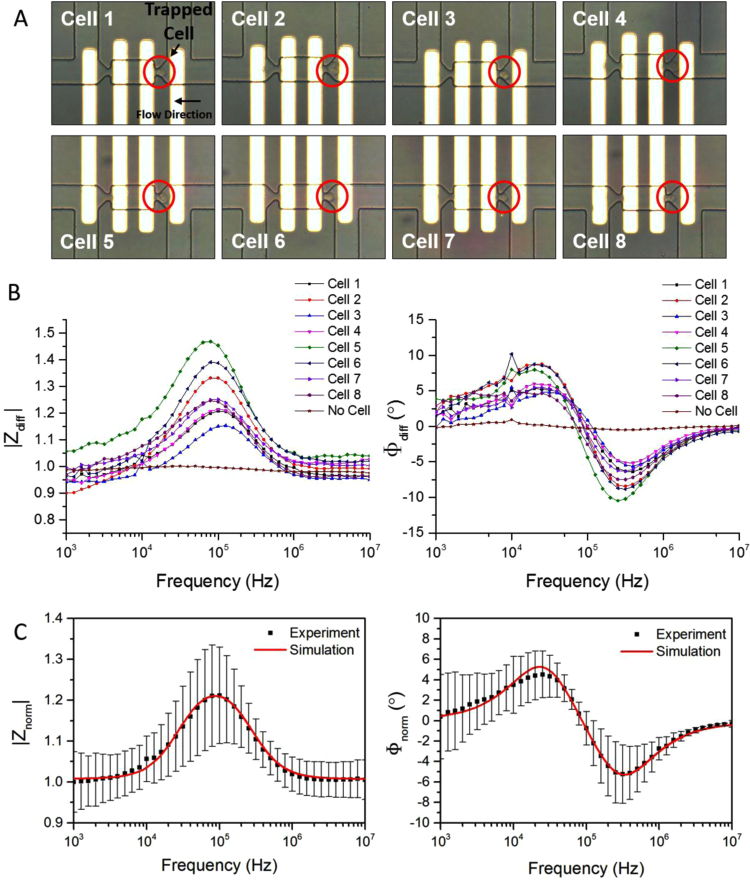
Single cell trapping and corresponding impedance spectrums. (A) Examples showing the trapping of fixed mouse embryonic stem cells. Flow direction was from right to left. Cells were captured in the right traps (sensing group), while the left traps were empty (reference group). (B) Differential impedance spectra of the eight single cells shown in (A), and an additional spectrum illustrating the case where no cell is trapped (i.e., both left and right traps are empty). ${\tilde{Z}}_{\mathrm{diff}}$ was obtained by normalising the impedance data from the sensing group to the impedance data from the reference group. Both Magnitude, $|Z_{\mathrm{diff}}$|, and the phase $\Phi_{\mathrm{diff}}$ are provided. (C) Average of the normalised impedance spectra of mESCs and curve fitting based on simulations. Both the magnitude and phase are presented. The experimental data point (black) shows the average value of ten cells. Error bars show the standard deviation. Simulations using MATLAB (red line) were based on the double-shell cell model. (For interpretation of the references to color in this figure legend, the reader is referred to the web version of this article.)

Single embryonic stem cells from mice were successfully trapped, and their impedance spectra were measured, analysed and fitted to physical models. Fig. 2A shows several examples of single cell trapping in the proposed device. Electrodes were patterned on the bottom glass substrate for impedance sensing. Fixed mouse embryonic stem cells were used in this experiment. The flow direction was from right to left, thus cells were captured in the traps facing to the right (upstream), while the traps facing to the left (downstream) were all kept empty. The traps where cells were captured are known as the sensing groups, and the empty traps located nearby are known as the reference groups. An input AC voltage was applied to the middle electrodes, and currents were measured from the rightmost and leftmost electrodes to calculate the impedance of the sensing group and the reference group respectively. Once a cell is trapped, it replaces the medium in the sensing volume, causing a change in the impedance across the sensing group, and thus the differential impedance spectrums, ${\tilde{Z}}_{\mathrm{diff}}$, which is obtained by normalising the impedance of the sensing group to that of the reference group. Fig. 2B shows the differential impedance spectrums, in terms of the magnitude ($|Z_{\mathrm{diff}}$|) and phase ($\Phi_{\mathrm{diff}}$), of the eight cells shown in Fig. 2A.

In the case where no cell is trapped (i.e., both left and right traps are empty), only the medium impedance is sensed in both the sensing group and the reference group. The impedance across the right trap should be the same as the impedance across the left trap due to the symmetric channel geometries. In ideal case, both the differential magnitude and phase spectrums should be straight lines (versus frequency). Fig. 2B shows such a spectrum, illustrating the case where no cell was trapped. The slight fluctuations of the straight-line response may result from the misalignment of the trapping channels with electrodes during fabrication process. Nevertheless, it can be seen from the figure that the differential impedance magnitude tends to be 1 and phase to be 0 across the whole frequency range when no cell is trapped.

To take into account the asymmetry problem caused by fabrication, all devices used in this work were calibrated prior to cell trapping and characterisation, serving as a baseline for further measurements. Define the baseline impedance as: ${\tilde{Z}}_{\mathrm{base}}=\frac{{\tilde{Z}}_{\mathrm{sen}\mathrm{se}}^{\prime }}{{\tilde{Z}}_{\mathrm{ref}}^{\prime }}$, where ${\tilde{Z}}_{\mathrm{sense}}^{\prime }$ and ${\tilde{Z}}_{\mathrm{ref}}^{\prime }$ are the impedance of the sensing group and the reference group before cell trapping (i.e., when no cell is trapped and only impedance of the medium is measured from both groups). In other words, ${\tilde{Z}}_{\mathrm{base}}$ is the response of the device itself. In order to eliminate the influence of device geometry mismatch and fabrication errors, the measured differential spectrum of a cell is normalized to the corresponding baseline spectrum, resulting in a normalised spectrum: ${\tilde{Z}}_{\mathrm{norm}}={\tilde{Z}}_{\mathrm{diff}}/{\tilde{Z}}_{\mathrm{base}}$ where ${\tilde{Z}}_{\mathrm{base}}$ corresponds to the value of ${\tilde{Z}}_{\mathrm{diff}}$ when no cell is trapped. The magnitude and phase of the normalised spectrum are $\left|{\tilde{Z}}_{\mathrm{norm}}\right|$ and $\Phi_{\mathrm{norm}}$, respectively. As the normalised spectrum minimises the measurement error caused by the device, this term would be used to describe the impedance spectrum of a cell throughout the text. ${\tilde{Z}}_{\mathrm{norm}}$ can be considered as an electrical signature of a particular cell and can be used to identify different cells.

Fig. 2C shows the averaged impedance spectrum of fixed mESCs and curve fitting based on the double-shell cell model. Both the normalised magnitude, $\left|{\tilde{Z}}_{\mathrm{norm}}\right|$, and phase, $\Phi_{\mathrm{norm}}$, are presented. Each experimental data point (black) shows the average value for ten cells while the error bar indicates the corresponding standard deviation. Variations in impedance among individual cells are caused by the intrinsic heterogeneity exhibited among cells. The intrinsic heterogeneity, defined as the cell-to-cell variability in the absence of inhomogeneity in the environment (Huang, 2009), is probably due to the fact that the cellular fluctuations are not synchronized between cells in the sample, i.e., cells are at different stages in a cell cycle and thus have different properties. The curve fitting was performed using Matlab (red line in Fig. 2C), by assuming that the following parameters are constant: $\sigma_{\mathrm{mem}}$= 8 µS/m; $\varepsilon_{\mathrm{cp}}$= 60$\varepsilon_{0}$; $\sigma_{\mathrm{ne}}$= 9.8 mS/m; $\varepsilon_{\mathrm{np}}$= 60$\varepsilon_{0}$; $d_{\mathrm{ne}}$= 40 nm; $\varepsilon_{0}$=8.854×10^–12^ F/m. The measured conductivity of cell suspending medium is 0.5 S/m. Electrical parameters of cells are extracted from the double-shell cell model and summarised in Table 1 (Fixed cells), in which the specific capacitance of membrane is described as: $C_{\mathrm{mem}}=\frac{\varepsilon_{\mathrm{mem}}}{d}$. The extracted parameter values are in general agreement with published literature, where cells were measured by impedance cytometry or dielectric spectroscopy (Asami et al., 1989, Ermolina et al., 2000, Holmes et al., 2009, Mansor and Ahmad, 2015, Polevaya et al., 1999). This work focuses on the study of mouse embryonic stem cells, and the extracted parameters applies to this particular cell line. Therefore, though the extracted values generally reside in the normal range reported in literature, they also exhibit difference, indicating the unique electrical properties of the mESCs being measured.

**Fig. 3:**
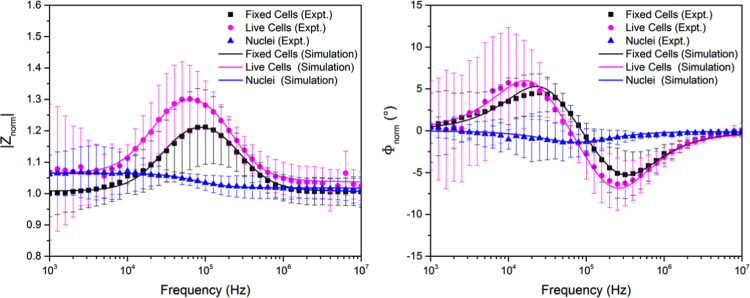
Experimental and simulation normalized impedance spectra for fixed cells, live cells and nuclei. Both the magnitude and phase are shown. Each experiment data point is the average value for ten cells. Error bar is the standard deviation. The experimental data of fixed cells and lived cells were fitted with the double-shell cell model, and the nuclei spectrum was fitted with single-shell model.

Single cell impedance measurements were performed for whole cells (both fixed and live cells) and for nuclei only. Fig. 3 summarised the averaged spectra of fixed/live cells and nuclei respectively. Each experimental data point indicates the average value of ten cells and the standard deviation is presented by the error bar. The experimental data of fixed and live cells were fitted to the double-shell cell model, while the nuclear spectrum was fitted to the single-shell model. Although the nuclear envelope has a double membrane structure, Asami et al. (Asami et al., 1989) had found that there was no difference between the two models when describing nuclei and only one dispersion was observed if the conductivity of the nuclear envelope is larger than 1 mS/m (which is usually the actual case). It was thus concluded that the nuclear envelope can be expressed by the single-shell model and this was employed to simplify the analysis. Extracted parameters for cells and nuclei are summarised in Table 1 and Table 2, respectively. A clear difference between whole cells and nuclei is observed from the impedance spectra. This is due to the fact that the dielectric properties of the nuclear envelope are significantly different from those of the cell membrane (Pethig et al., 2010). By extracting the corresponding electrical parameters for the nucleus from the experimental results, it is seen that the conductivity of the nuclear envelope (9.9 mS/m) is more than three orders of magnitude higher than that of the cell membrane (8.0 µS/m), providing experimental verification of the assumption used implicitly in previously published models (Asami et al., 1989, Ermolina et al., 2000, Pethig et al., 2010, Polevaya et al., 1999) that the nuclear envelop exhibits a much larger conductance compared with the cell membrane due to the existence of nuclear pores and ion channels in the nuclear envelope.

The difference in impedance spectra between fixed cells and live cells may result from the fixation process, during which the properties of cell membrane and cytoplasm may change. Even in the same sample, individual cells are seen to exhibit variations in electrical response. An explanation for the variation is that cells are at different cycle stages and hence have different sizes and dielectric properties. In addition to this, the change of cell shape and morphology during the trapping process, or the position where the cell is physically trapped can also be the reasons why the variations in cell impedance exit even in the same sample. Even though there are several factors that may influence the results, the averaged impedance spectra can still provide valuable and useful information for studying the behaviour of a particular cell population.

### 3.2 Study of stem cell differentiation

**Fig. 4:**
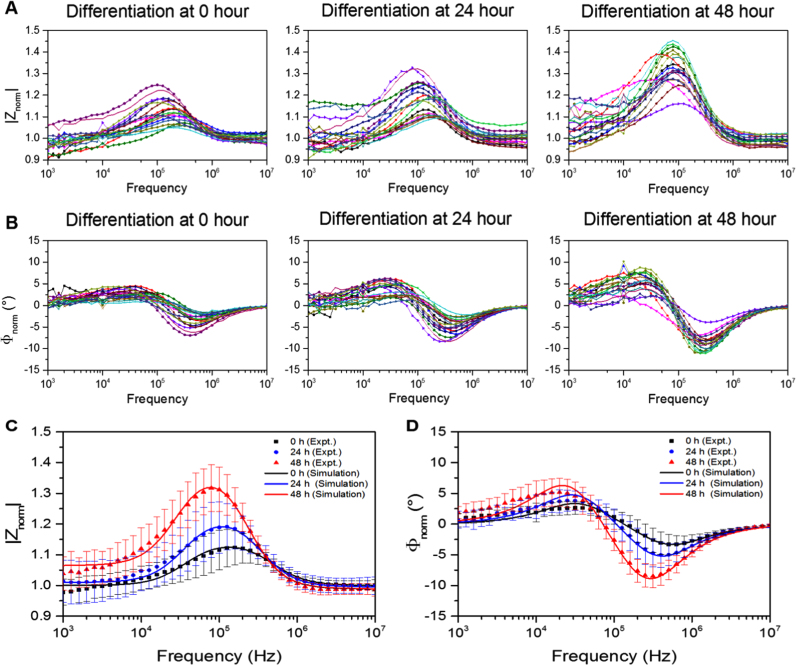
Cell differentiation study. (A) and (B) show the normalised impedance spectra (magnitude and phase respectively) of single cells at different differentiation states: 0 h, 24 h and 48 h. Each window shows spectra for eighteen individual cells overlaid in the plot. (C) and (D) present the averaged magnitude and phase data based on (A) and (B), as well as corresponding curve fittings. Each experimental data point shows the average value of 18 cells. Error bars show the standard deviation.

An impedance-based study of cell differentiation was carried out. Mouse embryonic stem cells were prepared and fixed at different time points in a differentiation cycle: 0 h (pre-differentiation); 24 h after differentiation; and 48 h after differentiation. Impedance spectra of single cells at different differentiation states are summarised in Fig. 4A (magnitude) and Fig. 4B (Phase). Eighteen cells were tested in each sample (0 h, 24 h and 48 h, respectively). A trend of impedance change is observed as the differentiation time increases. Cells differentiated at 48 h generally give more pronounced responses than those given by the undifferentiated cells (at 0 h). Most of the cells at 48 h have impedance spectra with large magnitudes, however, there was one cell that gives a much lower response. The impedance spectra for this cell seems to be similar with that of the undifferentiated cells (at 0 h), implying that this cell may not have commenced differentiation. Another interesting phenomenon is observed from cell sample differentiated at 24 h. The impedance spectra at 24 h tend to divide into two sub-groups, one with lower magnitudes similar to the undifferentiated cell at 0 h and the other one with higher magnitudes similar to the differentiated cells at 48 h, indicating that only part of the cells are differentiated while others remain undifferentiated. The impedance variation among individual cells, which is an indication of cell heterogeneity, is found to the largest when certain cells are found to be differentiated at 24 h. Undifferentiated (at 0 h) and fully differentiated (at 48 h) cells exhibit smaller heterogeneity compared with the cells in the transition state (at 24 h). The averaged impedance spectra of all cell samples are presented in Fig. 4C (magnitude) and Fig. 4D (Phase). Each experimental data point is the average value of eighteen cells. Error bar is the standard deviation. An increase in the impedance magnitude and phase (absolute value) is observed from cells differentiated at 0 h to cells differentiated at 48 h. The most sensitive frequency range to distinguish different cell samples, based on the magnitude spectrum, is from 10 kHz to 200 kHz. On the other hand, the frequency range where the phase data differ most is from 80 kHz to 1 MHz.

**Fig. 5:**
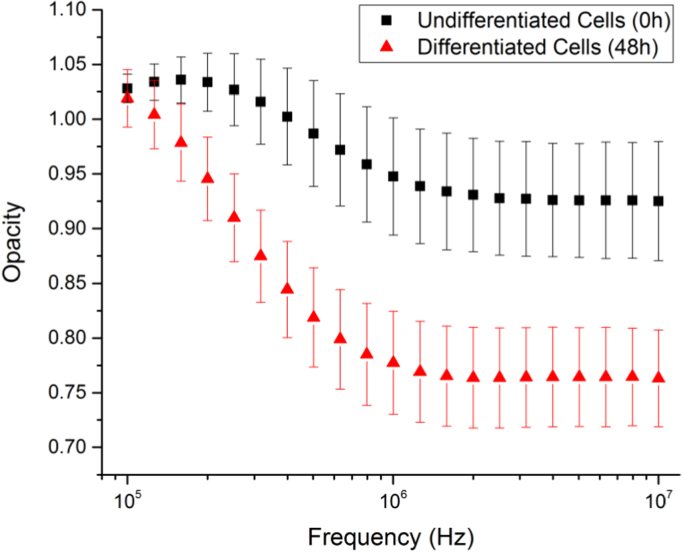
Measured opacity of undifferentiated cells (0 h) and differentiated cells (48 h) at frequencies ranging from 100 kHz to 10 MHz. The low frequency component is 50 kHz. Error bars present the standard deviations.

Opacity, which is defined as the ratio of the measured signal amplitude at high frequency to that at low frequency, has been widely used in cell impedance spectroscopy (Gawad et al., 2001, Holmes et al., 2009, Song et al., 2013). Song et al. had demonstrated a microfluidic impedance flow cytometer to distinguish the undifferentiated and differentiated cells (mouse embryonic carcinoma cell lines P19) by measuring the opacity at 1 MHz (vs. low frequency 50 kHz) (Song et al., 2013). In this work, opacity analysis was conducted for undifferentiated cells (0 h) and differentiated cell (48 h) in the frequency range of 100 kHz to 10 MHz. Here, the opacity is calculated by the taking the ratio of the normalised impedance at high frequency (${\tilde{Z}}_{\mathrm{norm\_highfreq}}$) to the normalised impedance at low frequency (${\tilde{Z}}_{\mathrm{norm\_lowfreq}}$). The low frequency component in the opacity term is measured at 50 kHz. Fig. 5 illustrates the opacity magnitude measured from 100 kHz to 10 MHz. It is observed that the two cell populations (0 h and 48 h) can be effectively distinguished by the opacity measured at frequencies higher than 200 kHz using the proposed device. In particular, a large difference between differentiated and undifferentiated cells at frequencies above 1 MHz has been observed. The finding is generally in agreement with the statement in the literature, however, the opacity difference between different cell populations measured in this work are more remarkable and distinguishable compared with other reported results (Song et al., 2013). The increase in sensitivity is contributed by the unique design of the device (e.g. the narrow trapping gap in the channel squeezes and concentrates current streamlines between sensing electrodes resulting in highest current density at the region where the cell is located; while the differential electrode configuration provides cancellation of common-mode drift).

Electrical parameters of cells at different differentiation states in Fig. 4 are extracted based on the double-shell cell model and summarised in Table 3. The following parameters are fixed: $\sigma_{\mathrm{mem}}$= 8 µS/m; $\varepsilon_{\mathrm{cp}}$= 60$\varepsilon_{0}$; $\sigma_{\mathrm{ne}}$= 9.8 mS/m; $\varepsilon_{\mathrm{ne}}$= 5$\varepsilon_{0}$; $d_{\mathrm{ne}}$= 40 nm; $\varepsilon_{\mathrm{np}}$= 60$\varepsilon_{0}$. The nucleus-to-cytoplasm (N/C) radius ratio is found to decrease during the differentiation process (83% for undifferentiated cells at 0 h and 67% for cells after 48 h differentiation). The decrease in N/C ratio with cell differentiation and maturity was also mentioned elsewhere (Arpitha et al., 2005, Pethig et al., 2010). An increase in cellular size but not nuclear size during embryonic stem cell differentiation has been demonstrated previously by high-resolution fluorescence microscopy (Pagliara et al., 2014). The authors have measured the cellular and nuclear cross sectional areas during embryonic stem cell differentiation and found that the cytoplasm increased significantly in cross-sectional area during differentiation (115 µm^2^ in cross-sectional area before differentiation and 150 µm^2^ after 48 h differentiation), while the nuclear cross-sectional area showed very little change (80 µm^2^ before differentiation and 82 µm^2^ after 48 h differentiation). Here, we have observed similar trends (which can be obtained from Table 3): the cytoplasm cross-sectional area shows an obvious increase during differentiation (104 µm^2^ before differentiation; 132 µm^2^ after 24 h differentiation; and 163 µm^2^ after 48 h differentiation), while the nuclear cross-sectional area almost remains constant (72 µm^2^ before differentiation; 72 µm^2^ after 24 h differentiation; and 73 µm^2^ after 48 h differentiation). The differentiation protocol used in this work is slightly different from the one used in the paper referenced (Pagliara et al., 2014), therefore, the absolute values stated here will not be identical to those stated in that paper. Nonetheless, the N/C ratio is found to decrease during cell differentiation.

On the other hand, the specific capacitance of the cell membrane is observed to increase during differentiation. This increase can be attributed to the increase in cell size. Other factors that might contribute to the increase of the specific cell membrane capacitance include a decrease in the thickness of the cell membrane or an increase in the permittivity of the cell membrane during differentiation. The research on investigating the biological mechanism behind these changes is still ongoing. There is an uncertainty in determining the conductivity of the cytoplasm and that of the nucleoplasm, as these two parameters have similar influences on the impedance spectrum. From the best fitting to the model, both the cytoplasm conductivity and nucleoplasm conductivity are found to increase during differentiation. However, due to the uncertainty in determining these two parameters, there could be other interpretations of this result. The biological explanation for the increase in cytoplasm conductivity or nucleoplasm conductivity is yet not very clear and is still under investigation. It should also be mentioned that the uncertainties associated with the measurement setup and the noise at high frequencies may influence the numerical fitting process when determining the conductivities of cytoplasm and nucleoplasm. Therefore, the standard deviations of the cytoplasm conductivity and nucleoplasm conductivity, stated in Table 3, comprise both the effects arising from cell-to-cell variations, and the additional uncertainties caused by the measurement system although these uncertainties are still much smaller than the actual change caused by cells. Nevertheless, the extracted parameters provide a quantitative indication of how the electrical properties of cells change during the differentiation process.

**Fig. 6:**
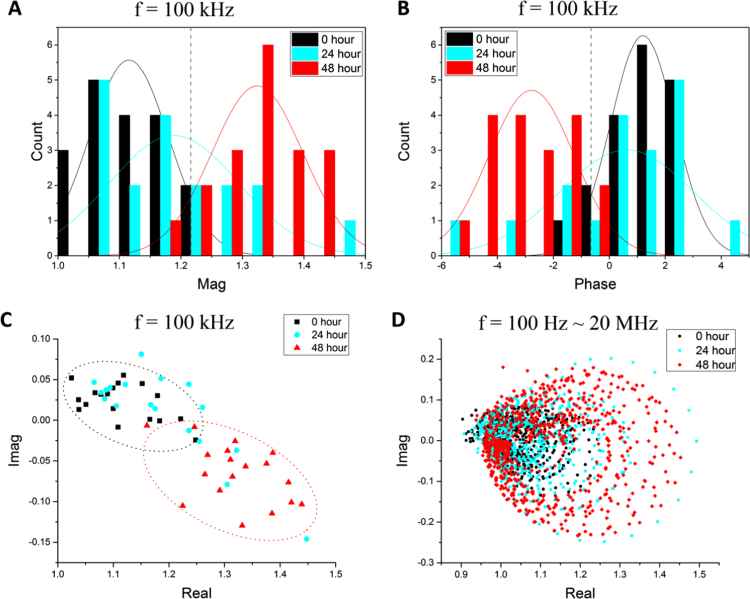
Statistical analysis of stem cell differentiation. (A) Histogram showing the impedance magnitude of 18 cells versus count number at the frequency of 100 kHz. (B) Histogram of impedance phase at the frequency of 100 kHz. (C) Real-imaginary scatter plot of cell impedance at 100 kHz. (D) Cole–cole plot (real-imaginary parts) showing the impedance of all cell samples (i.e., differentiated at 0 h, 24 h and 48 h) in the whole frequency range of 100 Hz –20 MHz. (For interpretation of the references to color in this figure legend, the reader is referred to the web version of this article.)

Fig. 6A and B illustrate the magnitude and phase histograms of cells measured at 100 kHz. The increase in the magnitude values for cells differentiated from 0 h to 48 h (Fig. 6A) mainly implicates the increase in cell volume that occurs during cell differentiation. As can be seen from Fig. 6B, the phase of the undifferentiated cells (0 h) is generally larger than 0 degrees at 10 kHz, whereas the phase of the differentiated cells (48 h) is less than 0 degrees. The different phase values for cells at different differentiation states can be attributed to the changes in the electrical properties of the cell membrane such as membrane conductivity and permittivity. The real-imaginary parts of the cell impedance at 100 kHz are presented as a scatter plot in Fig. 6C. In all graphs, the cells at 0 h (black) and at 48 h (red) are clearly distinguishable from each other. However, the data of the cells at 24 h (cyan) span across the whole magnitude/phase or real/imaginary range and exhibit pronounced heterogeneity, indicating that cells are at the transition state: part of the cells (roughly 30–40%) are differentiated whereas others are not. A statistical analysis (magnitude/phase histograms and real-imaginary scatter plot) was carried out for the impedance data acquired at all frequencies (see Supplementary Information Fig. S2, Fig. S3 and Fig. S4 for the analysis at selected frequencies). We found that the undifferentiated cells (at 0 h) and the fully differentiated cell (at 48 h) can be distinguished from each other in the frequency range from 10 kHz to 300 kHz from the magnitude histograms. Outside this rage, the two data sets overlap, and thus cannot be differentiated easily from the magnitude plots. On the other hand, the phase histograms of the cells at 0 h and 48 h start to show a difference at a relatively higher frequency (~ 80 kHz) compared with magnitude histograms, and the phase difference between the two samples disappears above 1 MHz (i.e., the two data sets merge together). Furthermore, from the real-imaginary scatter plots, the two populations (0 h and 48 h) can be distinguished over a wide frequency span, from 10 kHz up to 1 MHz, as these plots basically combine both the magnitude and phase information in one graph, i.e., Re(Z˜norm)=|Z˜norm|cos(Φnorm); $\mathrm{Imag}\left({\tilde{Z}}_{\mathrm{norm}}\right)=\left|{\tilde{Z}}_{\mathrm{norm}}\right|\sin(\Phi_{\mathrm{norm}})$. In summary, the most sensitive frequency regions to distinguish different types of cells are: 10–300 kHz from the magnitude histograms, 80 kHz–1 MHz from the phase histograms, and 10 kHz–1 MHz from the real-imaginary scatter plots.

Fig. 6D shows the impedance Cole-Cole plot, i.e., the real vs. imaginary part of the cell impedance over the whole frequency range from 100 Hz to 20 MHz, for all cell samples at 0 h, 24 h and 48 h. The Bode plots in Fig. 4 A and B show that cells at different differentiation states cannot be distinguished at very high or low frequency. This corresponds to the region in the Cole–Cole plot where the highest density of data points is observed and where the data sets of different samples overlap. Cells are distinguishable at a particular frequency range from the magnitude and phase information in the Bodes plots. Similarly, in the Cole–Cole plot, there is an area where the undifferentiated cells (0 h) and differentiated cells (48 h) exhibit a clear difference. Overall, the data points of undifferentiated cells at 0 h mostly concentrate in a relatively small area where the real parts range from 0.95 to 1.2 and the imaginary parts range from −0.1 to 0.1. On the other hand, the data points corresponding to the differentiated cell at 48 h span over a much larger circular area, with real parts ranging from 0.95 to 1.45 and imaginary parts from −0.25 to 0.2. Similar to the results shown in Bode plots, the data of cells differentiated at 24 h seem to cover the whole scope, overlapping the data range exhibited by cells at 0 h and 48 h.

The working hypothesis is that there must be a metastable transition state (at the time point of 24 h) during stem cell differentiation, from which ESCs have a choice to return to a naïve pluripotent state or irreversibly prime for differentiation (Pagliara et al., 2014). The transition could be used as a gateway to control differentiation and reprogramming. Cell population tends to exhibit a bimodal distribution (two distinct sub-populations) at the transition state during differentiation (Huang, 2009). We have observed in the impedance-based experiment that the degree of heterogeneity of a cell population is the highest when cells are characterised after 24 h (i.e., the variation in impedance spectrums among cells is the largest). The response of cells at 24 h tend to divide into sub-groups: some cells tend to have similar response compared to the undifferentiated cells (at 0 h), while others tend to be similar to cells characterised after 48 h. We hypothesise that the bimodality in impedance values would be more clearly observed as the sample size increases (i.e., with more cells being tested).

The proposed device has the benefit that not only the frequency response of an individual cell can be monitored, but also the averaged spectra of multiple cells representing a particular sample can be acquired and studied, due to the fact that a number of single cells can be efficiently and simultaneously trapped in the device. The impedance-based single cell study provides a quantitative analysis of cell properties. We have also performed initial experiments to verify the potential of using this device for long-term cell trapping and impedance sensing; however this requires the use of an incubator and external environmental control. Future work will investigate integrating these functions within the device to enable single cell studies within the device over a period of up to several days (Bell, 2011) together with associated recording of the impedance spectra.

## 4 Summary

A microfluidic device with integrated coplanar electrodes has been demonstrated for trapping and impedance sensing of individual cells. We have captured single mouse embryonic stem cells in the proposed device based on hydrodynamic trapping principles, monitored the impedance spectra corresponding to each individual cell and studied cell heterogeneity during differentiation using the impedance-based approach. Electrical parameters of stem cells are extracted by fitting theoretical models to experimental data. An increase in cell impedance has been found during cell differentiation. On the other hand, the nucleus-to-cytoplasm ratio tends to decrease during this process. The degree of heterogeneity of cells is observed to be the highest when the cells are at the transition state (24 h), compare with that at the undifferentiated (0 h) and fully differentiated (48 h) states. Not only can this device be used to provide label-free and non-invasive electrical parameter measurements of individual cells, but the device can also be used to study the heterogeneity of cells in a population, due to the efficient parallel single cell trapping and recording of impedance data. The proposed device can be adapted to monitor dynamic changes in electrical properties of individual cells over long periods of time by future integration of environmental (particularly temperature) control within the device.

## Figures and Tables

**Table 1:** Extracted parameters for fixed cells and live cells.

	Cell diameter (µm)	Nucleus-to-Cytoplasm (N/C) Radius Ratio	Membrane specific capacitance (F/m^2^)	Cytoplasm conductivity (S/m)	Nucleoplasm conductivity (S/m)
Fixed cells	13.40±1.24	0.72±0.07	0.026±0.004	0.48±0.05	1.02±0.10
Live cells	14.22±0.82	0.78±0.05	0.035±0.006	0.53±0.03	1.35±0.05

**Table 2:** Extracted parameters for nuclei.

Nucleus diameter (µm)	Nuclear envelope conductivity (mS/m)	Nuclear envelope specific capacitance (F/m^2^)	Nucleoplasm conductivity (S/m)
11.65±1.30	9.9±1.7	0.0015±0.0007	1.55±0.45

**Table 3:** Electrical properties of cells differentiated at 0 h, 24 h and 48 h, respectively.

Differentiation time (h)	Cell diameter (µm)	Nucleus-to-Cytoplasm (N/C) Radius Ratio	Membrane specific capacitance (F/m^2^)	Cytoplasm conductivity (S/m)	Nucleoplasm conductivity (S/m)
**0**	11.53±0.57	0.83±0.04	0.018±0.005	0.49±0.16	0.92±0.20
**24**	12.96±0.34	0.74±0.02	0.024±0.008	0.59±0.20	1.18±0.40
**48**	14.40±0.50	0.67±0.02	0.036±0.002	0.84±0.12	1.82±0.16
