# Supplementary material for: Single cell studies of mouse embryonic stem cell (mESC) differentiation by electrical impedance measurements in a microfluidic device
Source: Biosens Bioelectron. 2016 Jul 15;81:249–58. doi: 10.1016/j.bios.2016.02.069 (PMC4833703; doi:10.1016/j.bios.2016.02.069)
Supplement: Supplementary file 1 — Supplementary material [file mmc1.pdf]

Supplementary Information

Single cell studies of mouse embryonic stem cell (mESC) differentiation by electrical impedance measurements in a microfluidic device

Ying Zhou[a], Srinjan Basu[b], Ernest Laue[b], Ashwin A. Seshia[a,*]

[a] Nanoscience Centre, Department of Engineering, University of Cambridge, 11 JJ Thomson Avenue, Cambridge CB3 0FF, United Kingdom

[b] Department of Biochemistry, University of Cambridge, 80 Tennis Court Road, Cambridge CB2 1GA, United Kingdom

[*] Corresponding Author: Ashwin A. Seshia (aas41@cam.ac.uk)

Derivations of trapping channel dimensions

The width and total length of the bypass channel (i.e., Path 2) are indicated as W_2 and L_2. The total length of Path 1 is L_1, and Path 1 is composed of five regions (indicated as i, ii, iii, iv, v, in Fig. 1C). Each region in Path 1 has its own width and length: W_{11} and L_{11} for Region i; W_{12} and L_{12} for Region ii; W_{13} and L_{13} for Region iii; W_{11} and L_{14} for Region iv; W_{15} and L_{15} for Region v.

Pressure drop between A and B is $\Delta p = C(\alpha)L\frac{Q\mu(W+H)^2}{8(WH)^3}$, L is the length of the channel, Q is the volumetric flow rate, μ is the fluid viscosity, W and H is the width and height of the channel, α is the aspect ratio (W/H or H/W, $0 \leqslant \alpha \geqslant 1$), and $C(\alpha)$ is a constant which is a function of α, i.e., $C(\alpha) = 96(1 - 1.3553\alpha + 1.9467\alpha^2 - 1.7012\alpha^3 + 0.9564\alpha^4 - 0.2537\alpha^5)$.(Tan and Takeuchi 2007)

Since the Path 1 contain five regions as indicated in Fig. 1C, the total pressure drop across Path 1 can be expressed as:

$$\Delta p_1 = \Delta p_{11} + \Delta p_{12} + \Delta p_{13} + \Delta p_{14} + \Delta p_{15} \tag{S1}$$

where Δp_{1x} is the pressure drop at corresponding region in Path 1 (x denotes the region number).

$$\Delta p_{1x} = \mu C_{1x}(\alpha_{1x})L_{1x}Q_{1x}\frac{(W_{1x}+H)^2}{8(W_{1x}H)^3} \tag{S2}$$

The pressure drop in Path 2 is:

$$\Delta p_2 = \mu C_2(\alpha_2)L_2Q_2\frac{(W_2+H)^2}{8(W_2H)^3} \tag{S3}$$

Since the pressure drop between point A and B (in Fig. 1C) is constant, i.e., $\Delta p_1 = \Delta p_2$, we can get the following expression:

$$\frac{Q_2}{Q_1} = \frac{{W_2}^3}{C_2(\alpha_2)L_2(W_2+H)^2}\left[\sum_{x=1}^{5}\frac{C_{1x}(\alpha_{1x})L_{1x}(W_{1x}+H)^2}{(W_{1x})^3}\right] \quad \text{(S4)}$$

The design criterion for efficient particle trapping is that the volumetric flow rate along the bypass channel (Path 2) should be smaller than that of trapping path (Path 1),(Tan and Takeuchi 2007) i.e., $Q_2/Q_1 < 1$.

Therefore, the trapping criterion can be expressed as:

$$\frac{{W_2}^3}{C_2(\alpha_2)L_2(W_2+H)^2}\left[\sum_{x=1}^{5}\frac{C_{1x}(\alpha_{1x})L_{1x}(W_{1x}+H)^2}{(W_{1x})^3}\right] < 1 \quad \text{(S5)}$$

Depending on this criterion, we derived the dimensions of the channels and traps for the design in Fig. 1C. The geometric dimensions of these designed are summarised in Table S1.

Table S1: Channel dimensions (Unit: µm).

	$L_{11}(L_{14})$	$L_{12}(L_{15})$	W_{12}	W_{13}	L_{13}	W_2	L_2	H
5um Trapping Gap	10	5	5	25	50	25	805	15

Cell model

When an AC electric field is applied to a cell in suspension, the dielectric properties of the cell change as a function of frequency, known as Maxwell-Wagner dispersion. The dielectric behaviour of cell suspensions can be analysed using Maxwell's mixture theory. One basic model of a cell in suspension is the "single-shell" model (Fricke 1925), in which the cell membrane is modelled as a thin dielectric shell and the cytoplasm is modelled as a conducting homogeneous sphere. This model assumes only one spherical shell separating the cell inner from the ambient and also ignores the intracellular structures of cells thereby simplifying the analysis. The single-shell model showed a good agreement with experimental data for cells with negligible nucleus or organelles, however, it presented a partial disagreement with experiment for cells containing sizeable intracellular structures (Asami et al. 1989). To effectively accommodate the actual cell's morphology (e.g., the presence of a sizeable nucleus within the intracellular space), Irimajiri et al. proposed a more comprehensive cell analogy - the "double-shell" model (Irimajiri et al. 1978; Irimajiri et al. 1979). In this model, the cell is composed of four phases, i.e., cell membrane, cytoplasm, nuclear envelope and nucleoplasm (Fig. 1D). The "double-shell" implies the thin cell membrane and nuclear envelope, with the membrane separating the cytoplasm from the ambient medium and the nuclear envelope separating the nucleoplasm from

the cytoplasm. Each phase is modelled with its own electrical properties (conductivity 'σ' and permittivity 'ε'). The conductivity and permittivity of the medium are σ_{med} and ε_{med} respectively. The dielectric properties of the membrane and cytoplasm are significantly different from those of the nuclear envelope and nucleoplasm (Pethig et al. 2010). The double-shell model is more appropriate than the single-shell model for studying cells with a high nucleus-to-cytoplasm (N/C) ratio such as stem cells, as the properties of the nuclear envelope and nucleoplasm are taken into account.

Assuming a spherical cell is suspended in a medium, the equivalent complex permittivity of the whole mixture (cell and medium) is given by the Maxwell's mixture theory for heterogeneous systems:

$$\tilde{\varepsilon}_{mix} = \tilde{\varepsilon}_{med} \frac{2(1-\varphi) + (1+2\varphi)\, \tilde{\varepsilon}_{cell}/\tilde{\varepsilon}_{med}}{(2+\varphi) + (1-\varphi)\, \tilde{\varepsilon}_{cell}/\tilde{\varepsilon}_{med}} \tag{S6}$$

where $\tilde{\varepsilon}_{mix}$, $\tilde{\varepsilon}_{cell}$ and $\tilde{\varepsilon}_{med}$ are the complex permittivities of the cell-medium mixture, the cell and the suspending medium respectively, φ is the fractional volume of the cell relative to the suspending system. The complex dielectric permittivity is described in terms of the absolute permittivity (ε) and conductivity (σ), i.e., $\tilde{\varepsilon}(\omega) = \varepsilon - j\sigma$.

Based on the double-shell model (Fig. 1D), the equivalent complex permittivity of the whole cell, containing a spherical membrane, cytoplasm, nuclear envelope and nucleoplasm, can be written as:

$$\tilde{\varepsilon}_{cell} = \tilde{\varepsilon}_{mem} \frac{2(1-\varphi_1) + (1+2\varphi_1)\, \tilde{\varepsilon}_1/\tilde{\varepsilon}_{mem}}{(2+\varphi_1) + (1-\varphi_1)\, \tilde{\varepsilon}_1/\tilde{\varepsilon}_{mem}} \tag{S7}$$

where $\tilde{\varepsilon}_{mem}$ is the complex permittivity of the cell membrane, φ_1 is the fractional volume of cytoplasm sphere relative to the membrane sphere, i.e., $\varphi_1 = (\frac{R-d}{R})^3$. R and d are the outer radius of the cell and the thickness of the cell membrane, respectively. $\tilde{\varepsilon}_1$ is the equivalent complex permittivity of the inner part of the cell (including the cytoplasm, nuclear envelope and nucleoplasm) and is given by:

$$\tilde{\varepsilon}_1 = \tilde{\varepsilon}_{cp} \frac{2(1-\varphi_2) + (1+2\varphi_2)\, \tilde{\varepsilon}_2/\tilde{\varepsilon}_{cp}}{(2+\varphi_2) + (1-\varphi_2)\, \tilde{\varepsilon}_2/\tilde{\varepsilon}_{cp}} \tag{S8}$$

where $\tilde{\varepsilon}_{cp}$ is the complex permittivity of the cytoplasm. φ_2 is the volume fraction of the nucleus relative to the cytoplasm, i.e., $\varphi_2 = (\frac{R_{ne}}{R-d})^3$, where R_{ne} is the outer radius of the nuclear envelope. The equivalent complex permittivity of the nucleus (containing the nuclear envelop and nucleoplasm), $\tilde{\varepsilon}_2$, is given by:

$$\tilde{\varepsilon}_2 = \tilde{\varepsilon}_{ne} \frac{2(1-\varphi_3) + (1+2\varphi_3)\, \tilde{\varepsilon}_{np}/\tilde{\varepsilon}_{ne}}{(2+\varphi_3) + (1-\varphi_3)\, \tilde{\varepsilon}_{np}/\tilde{\varepsilon}_{ne}} \tag{S9}$$

where $\tilde{\varepsilon}_{ne}$, $\tilde{\varepsilon}_{np}$ are the complex permittivity of the nuclear envelope and nucleoplasm respectively, φ_3 is the fractional volume of the nucleoplasm sphere relative to the nuclear envelope sphere, i.e., $\varphi_3 = (\frac{R_{ne}-d_{ne}}{R_{ne}})^3$, and d_{ne} is the thickness of the nuclear envelope.

Fig. 1D presents the electrical model of a cell suspended in a medium inside a microfluidic system. The total complex impedance of a cell surrounded by a medium (i.e., cell-medium mixture) in the

sensing volume is $\tilde{Z}_{mix}$ and the complex impedance of the medium in the reference volume is $\tilde{Z}_{med}$. Coplanar electrodes are patterned on the bottom glass substrate. Between the electrode surface and the electrolyte forms an electrical double layer, of which the capacitance is notated as $\tilde{C}_{DL}$. With double layer capacitance taken into account, the total impedance measured from the electrodes in sensing group is $\tilde{Z}_{sense}$ and the total impedance measured from the electrodes in the reference group is $\tilde{Z}_{ref}$.

The complex impedance of the cell-medium mixture can be written as (Morgan et al. 2007):

$$\tilde{Z}_{mix} = \frac{1}{j\omega\tilde{C}_{mix}} \tag{S10}$$

where $\tilde{C}_{mix}$ is the complex capacitance of the cell-medium mixture.

For the coplanar electrode configuration, assuming the width and length of the coplanar electrodes are w and l respectively, and the spacing between them is $2g$, the complex capacitance of the cell-medium mixture between the two sensing electrodes can be derived using Schwartz-Christoffel transformation (Sun et al. 2007):

$$\tilde{C}_{mix} = \tilde{\varepsilon}_{mix} l \frac{K(\sqrt{1-k^2})}{K(k)} \tag{S11}$$

where $K(k)$ is the complete elliptic integral of the first kind, and k is given by:

$$k = \frac{\tanh(\frac{\pi g}{2h})}{\tanh(\frac{\pi(w+g)}{2h})}; \tag{S12}$$

The electrical double layer formed at the interface of the electrode surface and the electrolyte can affect the electrical response of the measurement system. The electrical double layer is modelled as a capacitor (C_{DL}), and is in series with the total complex impedance of the cell-medium mixture (Fig. 1D). Therefore, the total impedance measured from the sensing electrodes, $\tilde{Z}_{sense}$, can be expressed as:

$$\tilde{Z}_{sense} = \frac{2}{j\omega C_{DL}} + \tilde{Z}_{mix} \tag{S13}$$

For the reference group in Fig. 1D, the complex impedance of the medium between the two reference electrodes is given by:

$$\tilde{Z}_{med} = \frac{1}{j\omega\tilde{C}_{med}} \tag{S14}$$

where,

$$\tilde{C}_{med} = \tilde{\varepsilon}_{med} l \frac{K(\sqrt{1-k^2})}{K(k)} \tag{S15}$$

With double layer effects taken into account, the total impedance measured from the reference electrodes, $\tilde{Z}_{ref}$, is given by:

$$\tilde{Z}_{ref} = \frac{2}{j\omega C_{DL}} + \tilde{Z}_{med} \tag{S16}$$

The differential spectrum of a cell can be obtained by normalising the impedance of the sensing group with regard to the impedance of the reference group (Malleo et al. 2010):

$$\tilde{Z}_{diff} = \frac{\tilde{Z}_{sense}}{\tilde{Z}_{ref}} \tag{S17}$$

where the magnitude, $|\tilde{Z}_{diff}|$, and the phase, Φ_{diff}, of the differential impedance spectrum are,

$$|\tilde{Z}_{diff}| = \frac{|\tilde{Z}_{sense}|}{|\tilde{Z}_{ref}|} \; ; \quad \Phi_{diff} = \Phi_{diff} - \Phi_{ref} \tag{S18}$$

Device fabrication details

Electrode fabrication. Electrodes were patterned on 2” Pyrex glass wafers by lift-off. AZ 5214E photoresist (MicroChemicals) was used in image reversal mode as the sacrificial layer for the lift-off process. First, the glass substrate was cleaned and dehydrated on a 200°C hot plate for 5 minutes. Photoresist spin coating was performed immediately after cooling down the substrate to avoid re-adsorption of water. AZ 5214E was spin-coated onto the wafer at 500 rpm for 5 seconds and then 4000 rpm for 45 seconds. The resulting film thickness was 1.6 um, calibrated by profilometer. After removing the edge bead by acetone, the substrate was pre-baked at 105°C for 60 seconds, and then rehydrated at room temperature for 10 minutes before exposure to allow a certain water content to be present in the resist during exposure, so that a reasonably high development rate and contrast could be achieved. UV exposure of the resist was done with the Karl Suss MJB4 mask aligner (exposure energy: 10 mJ/cm^2). After exposure, the sample was relaxed for 10 minutes to outgas the nitrogen formed during exposure from the exposed resist. Reversal bake of the resist was performed on a 116°C hotplate for 60s, after which a flood UV exposure was done using the mask aligner (exposure energy: 300 mJ/cm^2). The resist was developed in AZ 726 MIF developer (MicroChemicals) for 90 seconds, rinsed with DI water and blow-dried with nitrogen gas. The resist was slightly over-developed to create undercut and ease the lift-off process. Titanium (20 nm, as an adhesion layer) and gold (100 nm) were deposited on the sample by electron beam evaporation (Kurt J. Lesker e-Beam Evaporator PVD 75) at a deposition rate of 1Å/s. After metal deposition, the photoresist was removed in AZ 100 remover bath at 50°. After lift-off, the substrate was thoroughly rinsed with DI water and dried with nitrogen, and electrode patterns were inspected with microscope. The width of the electrodes at the sensing region of the chip is 15 µm, and the distance between two neighbouring electrodes is 15 µm.

Master wafer fabrication. The master moulds for the microfluidic trapping channels (geometric dimensions are listed in Table S1) were fabricated using negative photoresist SU-8 2015 (MicroChem) by standard photolithography process. SU-8 2015 was spin-coated onto a 3” silicon wafer at 500 rpm for 8 seconds and 3000 rpm for 60 seconds, resulting in a 15µm-thick film. Soft bake was performed at 65°C for 1 minute and 95°C for 4 minutes. Exposure was done with Karl Suss MJB4 mask aligner (exposure energy: 140 mJ/cm^2). After exposure, the wafer was post baked at 65°C for 1 minute, 95°C

for 3 minutes. After cooling down, the resist was then developed in SU-8 developer (PGMEA) for 3 minutes (with gentle agitation), then rinsed thoroughly with propanol and dried with nitrogen. A hard bake step was performed at 200°C for 5 minutes to improve the mechanical properties and thermal performance of SU-8. Prior to PDMS moulding, the surface of the master wafer was rendered hydrophobic by vapour deposition of FDTS (1H,1H,2H,2H-Perfluorodecyltrichlorosilane, 96%, Alfa Aesar).

PDMS rapid prototyping. PDMS (Sylgard 184 silicone elastomer kit, Dow Corning) was prepared by mixing 10:1 base to curing agent. The mixture was degassed in a vacuum desiccator for 1 hour to remove all air bubbles. After degassing, the mixture was spun onto the master mould at 500 rpm for 8 seconds and then 1000 rpm for 60 s. This layer was cured on a 65°C hot plate for 2 minutes and on a 150°C hot plate for 5 minutes. The resulting PDMS layer (~ 60 um thickness) was thin enough that the shrinkage of PDMS, caused thermal expansion and contraction during the curing process, can be neglected. We found that this approach can effectively solve the shrinkage-induced PDMS registration problem during the alignment and bonding process of microfluidic channels and electrode patterns. A separate thick PDMS block, serving as a substrate for the thin PDMS layer for easy handling, was made by mixing the base and curing agent with 10: 1 ratio, degassing for 1 hour and then pouring onto a blank wafer. After curing, the thick PDMS substrate was peeled-off from the blank wafer, and bonded to the thin PDMS layer immediately after oxygen plasma treatment (Diener etcher, 100% power for 20 seconds at 1.0 mbar). The bonded sample was further baked in a 65°C oven for 1 hour to improve the bonding strength, after which the PDMS was peeled off from the master mould and cut into desired shape. After channel inlets/outlets (1 mm diameter) were drilled using a biopsy punch, the PDMS sample was ready for bonding with glass to close the channel. Before bonding, the PDMS sample (containing microfluidic channels) and the glass substrate (containing electrode patterns) were cleaned properly and activated by oxygen plasma. Immediately after the plasma activation, the two pieces were aligned and brought into conformal contact to form permanent bonding. A soft force was applied to remove any trapped air bubbles. The bonded sample was then baked in a 65°C oven for 1 hour to improve the bonding strength.

Device assembling and experiment setup. Surface mount connectors (0.1" surface mount terminal strip, Samtec TSM series) were used to make the connections between the electrode patterns on the fabricated chip and external measuring instrumentation. The connectors were bonded to the electrode pads on the chip using silver conductive epoxy (Fig. 1A). The microfluidic device was connected to a 1 ml syringe with PTFE tubing (0.59mm ID x 0.25mm Wall) and 23G needles. Fluid flow was controlled by syringe pumps. Before use, the channels were pre-treated with 1% BSA (in 1×PBS) for 30 minutes to block hydrophobic interactions between biological samples and PDMS surface. The chip was connected with an impedance analyser (Solartron SI 1260) for impedance measurements. Prior to cell characterisation, the device was first filled with PBS buffer, and a calibration experiment was performed for the device itself, serving as a baseline for further cell measurements. After the calibration, cells were loaded into the device at flow rate of 20 µl/hr. Once all traps were occupied by cells (inspected with microscope), the device was washed with buffer. Cell impedance measurements were then conducted with the impedance analyser. A 100 mV input single was used. The frequency range was from 100 Hz to 20 MHz, with 10 points being measured per decade. Analytical simulations based on the double-shell cell model were performed using Matlab. Numerical simulations were conducted using COMSOL 4.4 (AC/DC module).

Fig. S1

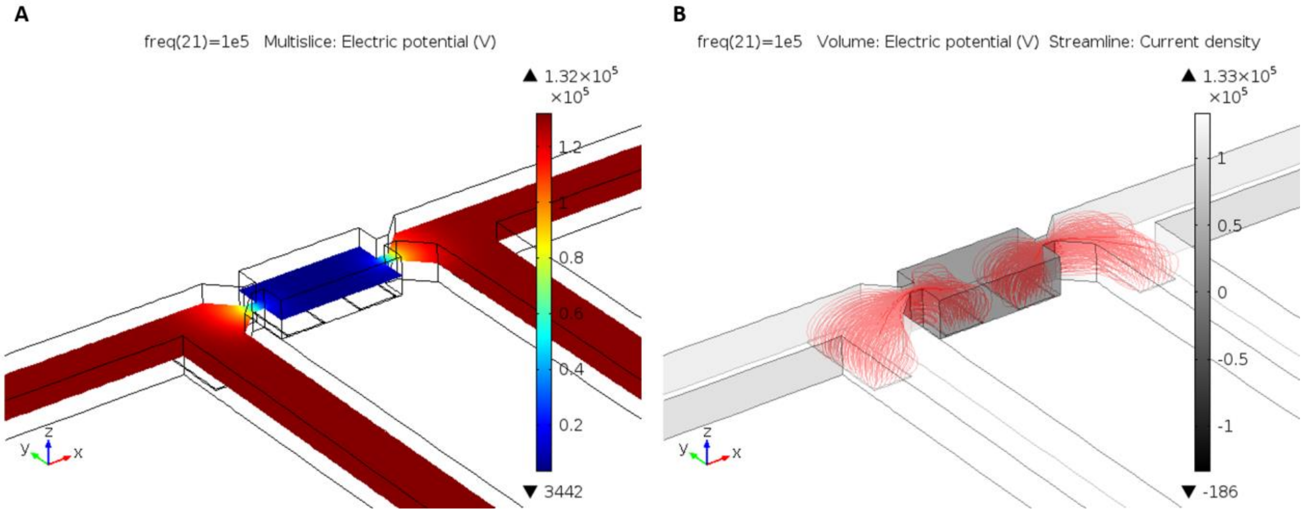

Fig. S1: Simulations showing the electrical filed distribution and current density inside the channel. Four electrodes are patterned on the bottom channel wall. Simulations are performed using COMSOL Multiphysics 4.4 AC/DC module. (A) Electric potential distribution in the channel at 100 kHz. Only the middle plane in z direction is shown for clarity. (B) Streamlines showing the current density inside the channel at 100 kHz. Current streamlines are squeezed and concentrated via the narrow trapping gap, resulting in highest current density through the trapping gap.

Fig. S2

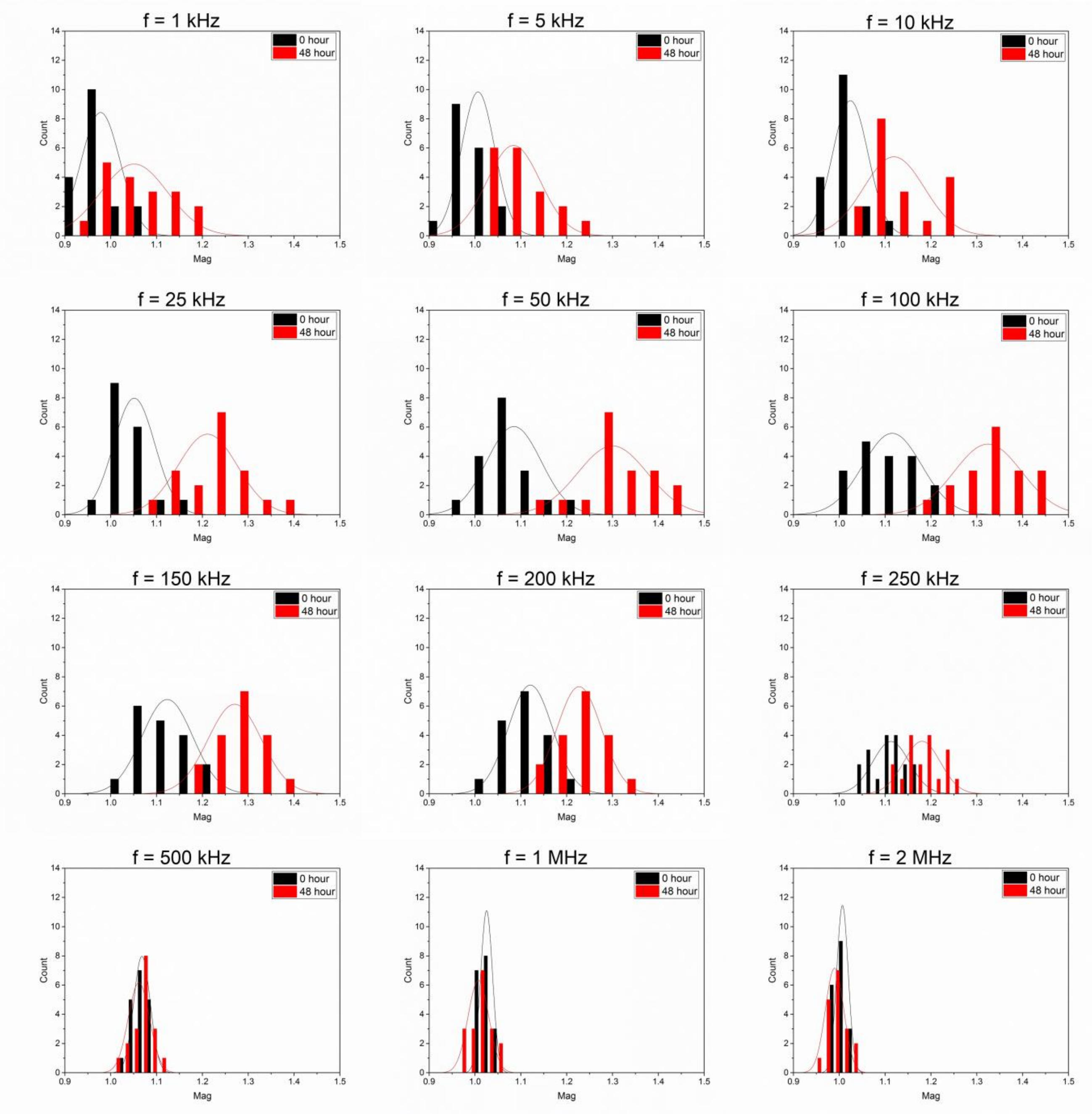

Fig. S2: Magnitude histograms at different frequencies (cell differentiation experiment).

Fig. S3

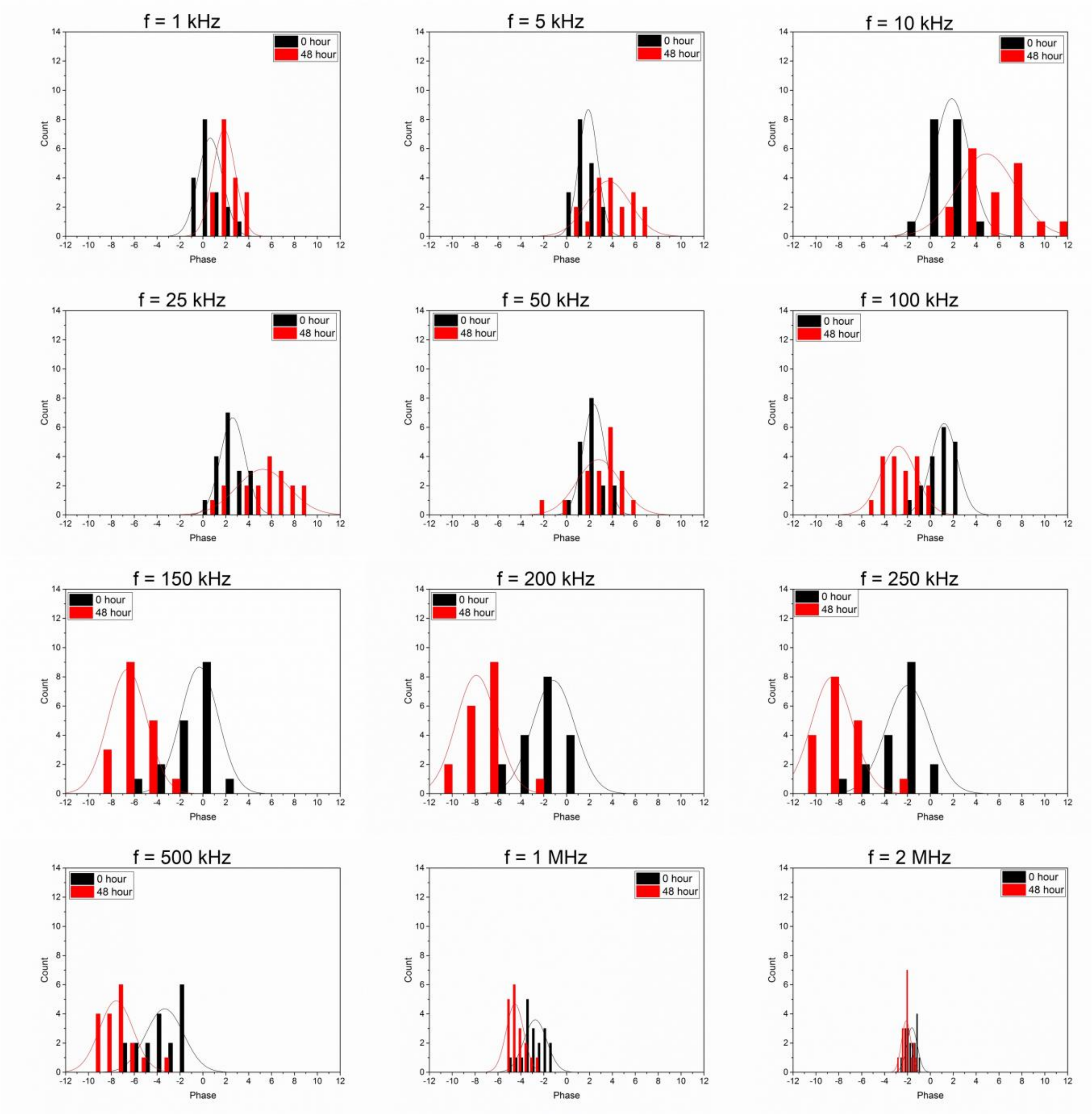

Fig. S3: Phase histograms at different frequencies (cell differentiation experiment).

Fig. S4

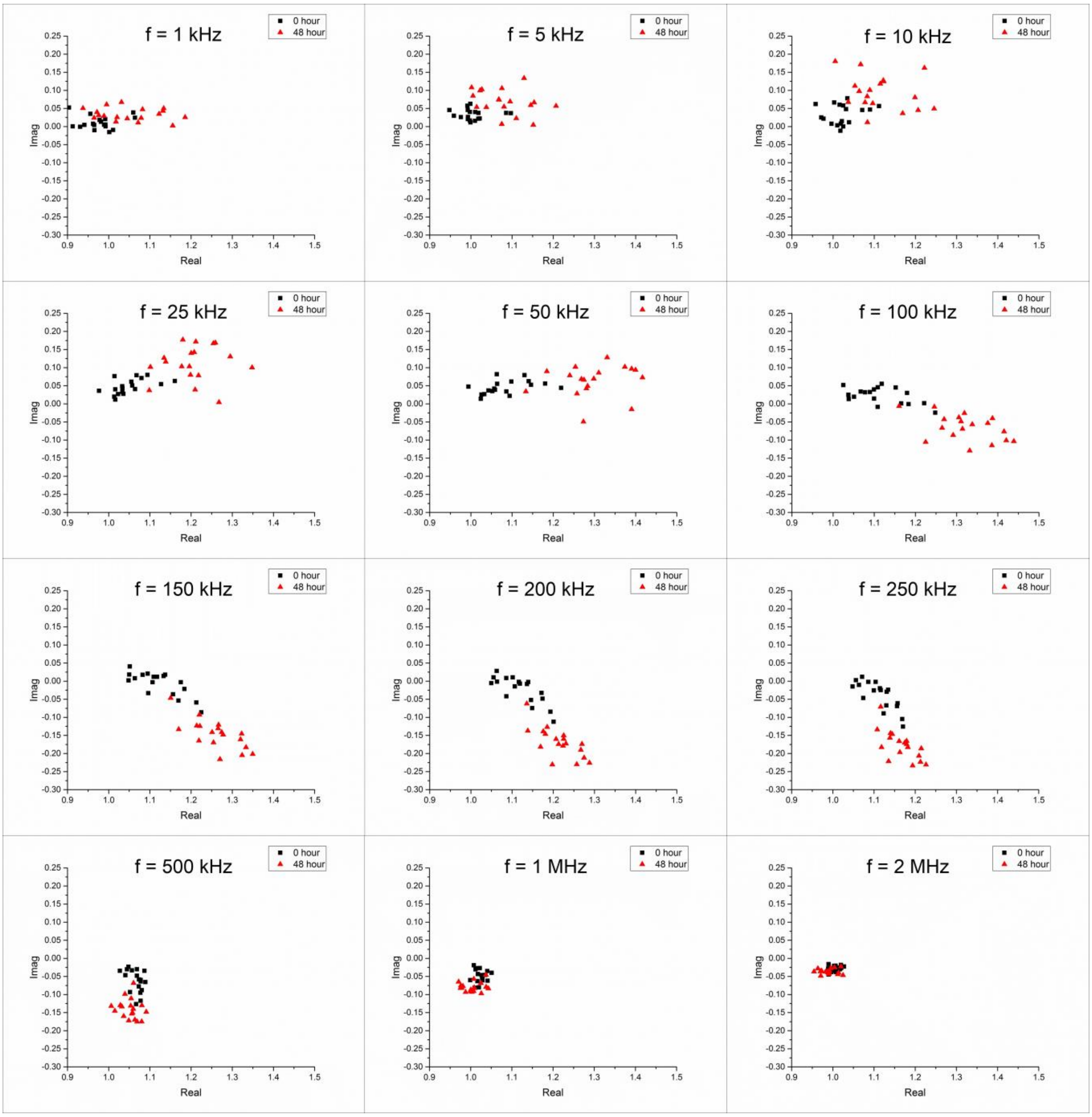

Fig. S4: Real-imaginary impedance scatter plots at different frequencies (cell differentiation experiment).

References

Asami, K., Takahashi, Y., Takashima, S., 1989. Dielectric Properties of Mouse Lymphocytes and Erythrocytes. Biochim. Biophys. Acta 1010(1), 49-55.

Fricke, H., 1925. A Mathematical Treatment of the Electric Conductivity and Capacity of Disperse Systems ii. The Capacity of a Suspension of Conducting Spheroids Surrounded by a Non-Conducting Membrane for a Current of Low Frequency. Phys. Rev. 26(5), 678-681.

Irimajiri, A., Doida, Y., Hanai, T., Inouye, A., 1978. Passive electrical properties of cultured murine lymphoblast (L5178Y) with reference to its cytoplasmic membrane, nuclear envelope, and intracellular phases. J. Membr. Biol. 38(3), 209-232.

Irimajiri, A., Hanai, T., Inouye, A., 1979. Dielectric Theory of Multi-Stratified Shell-Model with Its Application to a Lymphoma Cell. J. Theor. Biol. 78(2), 251-269.

Malleo, D., Nevill, J.T., Lee, L.P., Morgan, H., 2010. Continuous differential impedance spectroscopy of single cells. Microfluid. Nanofluid. 9(2-3), 191-198.

Morgan, H., Sun, T., Holmes, D., Gawad, S., Green, N.G., 2007. Single cell dielectric spectroscopy. Journal of Physics D-Applied Physics 40(1), 61-70.

Pethig, R., Menachery, A., Pells, S., De Sousa, P., 2010. Dielectrophoresis: a review of applications for stem cell research. J. Biomed. Biotechnol. 2010, 182581.

Sun, T., Green, N.G., Gawad, S., Morgan, H., 2007. Analytical electric field and sensitivity analysis for two microfluidic impedance cytometer designs. Iet Nanobiotechnol 1(5), 69-79.

Tan, W.H., Takeuchi, S., 2007. A trap-and-release integrated microfluidic system for dynamic microarray applications. Proc. Natl. Acad. Sci. U. S. A. 104(4), 1146-1151.
